# Automated machine learning (AutoML) can predict 90-day mortality after gastrectomy for cancer

**DOI:** 10.1038/s41598-023-37396-3

**Published:** 2023-07-08

**Authors:** Gopika SenthilKumar, Sharadhi Madhusudhana, Madelyn Flitcroft, Salma Sheriff, Samih Thalji, Jennifer Merrill, Callisia N. Clarke, Ugwuji N. Maduekwe, Susan Tsai, Kathleen K. Christians, T. Clark Gamblin, Anai N. Kothari

**Affiliations:** 1 Medical Scientist Training Program, Medical College of Wisconsin, Milwaukee, USA; 2 Division of Surgical Oncology, Department of Surgery, Medical College of Wisconsin, 8701 Watertown Plank Road, Milwaukee, WI 53226 USA

**Keywords:** Surgical oncology, Prognosis, Risk factors

## Abstract

Early postoperative mortality risk prediction is crucial for clinical management of gastric cancer. This study aims to predict 90-day mortality in gastric cancer patients undergoing gastrectomy using automated machine learning (AutoML), optimize models for preoperative prediction, and identify factors influential in prediction. National Cancer Database was used to identify stage I–III gastric cancer patients undergoing gastrectomy between 2004 and 2016. 26 features were used to train predictive models using H2O.ai AutoML. Performance on validation cohort was measured. In 39,108 patients, 90-day mortality rate was 8.8%. The highest performing model was an ensemble (AUC = 0.77); older age, nodal ratio, and length of inpatient stay (LOS) following surgery were most influential for prediction. Removing the latter two parameters decreased model performance (AUC 0.71). For optimizing models for preoperative use, models were developed to first predict node ratio or LOS, and these predicted values were inputted for 90-day mortality prediction (AUC of 0.73–0.74). AutoML performed well in predicting 90-day mortality in a larger cohort of gastric cancer patients that underwent gastrectomy. These models can be implemented preoperatively to inform prognostication and patient selection for surgery. Our study supports broader evaluation and application of AutoML to guide surgical oncologic care.

## Introduction

Although the incidence of gastric cancer is decreasing, it remains the fourth leading cause of cancer-related death world-wide^1^. Surgery is the only curative treatment; however, postoperative mortality rates remain high with a 90-day mortality of 9.1% following total gastrectomy^2^. The risk of significant perioperative morbidity makes the decision for proceeding with gastric resection challenging in some patients. Studies have shown that aggressive treatments and delayed hospice care can decrease quality of life for patients with advanced cancers and their families^3,4^, and thus accurate early mortality risk prediction following gastrectomy is crucial for clinical management of gastric cancer.

Machine learning (ML) has the potential to more accurately make predictions when compared to traditional statistical methodologies, as it is designed to capture multifaceted non-linear relationships and complex interactions between variables^5,6^. ML has been applied to improve prognostication in various disease states^6^; within gastric cancer specifically, ML models have been shown to improve endoscopic/pathology-based diagnosis^7^ and predict postoperative disease recurrence^8,9^ and lymph node metastasis^10^. Despite its advantages, some criticisms of ML include difficulty with selecting and training appropriate models, managing a complex set of input features and pre-processing data, and scaling fitted models to production^6^. Moreover, despite the availability of extensive data within electronic health records, the need for expertise in ML has been proposed to be one of the major factors limiting the widespread application of ML models in healthcare^11^.

Automated Machine learning (AutoML) is an emerging field within ML that provides user-friendly tools for training high quality, scalable models and decreases the reliance on human experts^11^. Numerous open-source and industry-produced AutoML tools have been developed in recent years; however, their application to clinical prediction have been limited^11^. H2O.ai’s AutoML^12^ is a freely available, easy-to-use interface that allows users to train a variety of pre-developed candidate models. It has also been reported to have improved performance^13^ and more versatile features compared to other AutoML tools^14^. The primary objective of this study was to assess whether AutoML can predict 90-day mortality in patients with gastric cancer undergoing gastric resection. Secondary objectives included optimizing models for preoperative prediction and identifying factors that most strongly contribute to predictions of mortality after gastric cancer surgery.

## Materials and methods

The National Cancer Database (NCDB) was used to identify stage I–III gastric cancer patients undergoing gastrectomy between 2004 and 2016. The NCDB is a hospital-based cancer registry developed by the American College of Surgeons Commission on Cancer (CoC) and the American Cancer Society. Data are made available publicly to investigators associated with a CoC-accredited cancer program. It includes data from over 1500 CoC-accredited programs and captures approximately 70% of patients with new cancer diagnoses in the United States^15^. Patients with metastatic disease and palliative-intent surgery were excluded. 26 input features were selected and used to predict 90-day mortality (Table 1). Characteristics of patients alive at 90 days versus those that died were compared using two-tailed t-tests or chi-square analysis for continuous and categorical variables respectively. IBM SPSS Version 28.0 was utilized for descriptive statistical analyses. This study was reviewed and approved by the Medical College of Wisconsin Institutional Review Board with waiver of informed consent (retrospective study with non-identifiable patient records) and conducted in accordance with relevant guidelines and regulations.

**Table 1:** Preoperative features of stage I–III gastric cancer patients undergoing gastrectomy between 2004 and 2016.

Variables	All patients (N = 39,108)	Alive at 90 days (N = 35,635)	90-day mortality (N = 3473)	*p*-value
Age (Mean ± Std. Deviation)	67.61 ± 12.21	67.04 ± 12.174	73.5 ± 10.9	< 0.0001
Sex				0.276
Female	12,728 (33%)	11,569 (32%)	1159 (33%)	
Male	26,380 (68%)	24,066 (68%)	2314 (67%)	
Partial gastrectomy	10,669 (27%)	9655 (27%)	1014 (29%)	0.008
Distal gastrectomy	8160 (21%)	7501 (21%)	659 (19%)	0.004
Total gastrectomy	16,352 (42%)	15,000 (42%)	1352 (39%)	< 0.001
En bloc gastrectomy	3927 (10%)	3479 (10%)	448 (13%)	< 0.001
Charlson-Deyo score				< 0.001
0	25,075 (64%)	23,083 (65%)	1992 (57%)	
1	9864 (25%)	8950 (25%)	914 (26%)	
2	2945 (8%)	2567 (7%)	378 (11%)	
3	1224 (3%)	1035 (3%)	189 (5%)	
Days from diagnosis to treatment (Mean ± Std. Deviation)	32.18 ± 32.37	32.79 ± 32.51	25.9 ± 30.2	< 0.0001
Length of surgical inpatient stay, in days (Mean ± Std. Deviation)	11.7 ± 10.8	11.3 ± 10.5	15.85 ± 13.56	< 0.0001
Neoadjuvant radiation therapy	6132 (16%)	5769 (16%)	363 (10%)	< 0.001
Neoadjuvant chemotherapy	8011 (21%)	7521 (21%)	490 (14%)	< 0.001
AJCC clinical T				< 0.001
1	5864 (15%)	5513 (15%)	351 (10%)	
2	4959 (13%)	4615 (13%)	344 (10%)	
3	8809 (23%)	8226 (23%)	583 (17%)	
4	1395 (4%)	1206 (3%)	189 (5%)	
5	18,081 (46%)	16,075 (45%)	2006 (58%)	
AJCC clinical N				< 0.001
0	16,634 (43%)	15,438 (43%)	1196 (34%)	
1	22,474 (58%)	20,197 (57%)	2277 (66%)	
Grade				< 0.001
Cell type not determined, not stated or not applicable, unknown primaries, high grade dysplasia	2314 (6%)	2166 (6%)	148 (4%)	
Moderately differentiated, moderately well differentiated, intermediate differentiation	13,430 (34%)	12,342 (35%)	1088 (31%)	
Poorly differentiated	20,158 (52%)	18,159 (51%)	1999 (58%)	
Undifferentiated, anaplastic	572 (2%)	497 (1%)	75 (2%)	
Well differentiated, differentiated, NOS	2634 (7%)	2471 (7%)	163 (5%)	
Tumor size (mm; Mean ± Std. Deviation)	65.61 ± 128.46	64.26 ± 125.86	79.41 ± 151.99	< 0.001
Node ratio (Mean ± Std. Deviation)	0.19 ± 0.28	0.176 ± 0.270	0.323 ± 0.364	< 0.0001
RACE				< 0.001
White	29,067 (74%)	26,313 (74%)	2754 (79%)	
Black	5772 (15%)	5267 (15%)	505 (15%)	
American Indian, Aleutian, or Eskimo	3445 (9%)	3268 (9%)	177 (5%)	
Chinese	824 (2%)	787 (2%)	37 (1%)	
Hispanic	5727 (15%)	5237 (15%)	490 (14%)	0.35
Insurance				< 0.001
Not insured	2002 (5%)	1850 (5%)	152 (4%)	
Private insurance/managed care	12,938 (33%)	12,245 (34%)	693 (20%)	
Medicaid	2446 (6%)	2291 (6%)	155 (4%)	
Medicare	21,722 (56%)	19,249 (54%)	2473 (71%)	
Medicaid expansion	25,111 (64%)	22,906 (64%)	2205 (63%)	0.354
URBAN	21,559 (55%)	19,789 (56%)	1770 (51%)	< 0.001
LOCATION				< 0.001
New England	17,426 (45%)	15,944 (45%)	1482 (43%)	
Middle Atlantic	14,504 (37%)	13,077 (37%)	1427 (41%)	
South Atlantic	7178 (18%)	6614 (19%)	564 (16%)	
Median household income for each patient’s area of residence				< 0.001
< $38,000	7517 (19%)	6785 (19%)	732 (21%)	
≥ $63,000	12,263 (31%)	11,316 (32%)	947 (27%)	
$38,000–$47,999	8787 (23%)	7899 (22%)	888 (26%)	
$48,000-$62,999	10,342(26%)	9454 (27%)	888 (26%)	
Measure of educational attainment for each patient's area of residence				0.001
< 7.0%	8157 (21%)	7511 (21%)	646 (19%)	
> = 21.0%	8414 (22%)	7659 (21%)	755 (22%)	
13.0–20.9%	10,194 (26%)	9207 (26%)	987 (28%)	
7.0–12.9%	12,160 (31%)	11,092 (31%)	1068 (31%)	
Residence to Hospital Distance (miles; Mean ± Std. Deviation)	34.14 ± 113.12	35.03 ± 116.72	25.01 ± 64.85	< 0.001
FACILITY TYPE				< 0.001
Academic/Research Program (includes NCI-designated comprehensive cancer centers)	17,472 (45%)	16,293 (46%)	1179 (34%)	
Community Cancer Program	2349 (6%)	2030 (6%)	319 (9%)	
Comprehensive Community Cancer Program	13,208 (34%)	11,786 (33%)	1422 (41%)	
Integrated Network Cancer Program	5374 (14%)	4842 (14%)	532 (15%)	
Not available	705 (2%)	684 (2%)	21 (1%)	

Characteristics of patients alive at 90 days versus those that died were compared using two-tailed t-tests or chi-square analysis for continuous and categorical variables respectively.

The H2Oai’s AutoML^16^ package for RStudio was utilized to train 20 ML algorithms that were either linear, decision tree-, or neural network- based. While the linear models are most suited for characterizing linear relationship, the decision trees are better suited for multi-level categorical variables (i.e. yes/no decisions), and the neural networks can best handle complex variable interactions^17^. Stacked ensembles, which are a combination of the trained models, were also generated. Data were split into training and validation sets. fivefold cross-validation was used during model training. Model performance of the top ensemble and top independent model type on the validation set was evaluated using area under the receiver operating characteristic curve (AUC), positive and negative predictive values as well as sensitivity and specificity. To do so, a binary classifier based on 90-day mortality was generated and performance measured based on an F1-threhold optimized for specificity and negative predictive value. Shapley additive explanations plots (SHAP), variable importance heatmaps, and partial dependence plots were generated for model interpretability. Detailed documentation as well as directions for implementation of H2O.ai are freely available online^16^.

### Conference presentation

Society of Alimentary Tract Annual Meeting 2022, San Diego, California.

## Results

39,108 patients with gastric cancer that underwent gastrectomy for gastric cancer between 2004 and 2016 were included in the study (Table 1). Of those patients, 3473 (8.8%) died within 90 days postoperatively. There was a greater proportion of males than females (67.5% vs. 32.5%) in the study, with no significant differences in sex among patients who were and were not alive at 90 days post-surgery. Compared to the patients who were alive 90 days postoperatively, those who died were older (73.5 ± 10.9 years vs. 67.04 ± 12.17 years, *p* < 0.001), had longer postoperative hospital length of stay (15.85 ± 13.56 days vs. 11.3 ± 10.5, *p* < 0.001), had a shorter duration between diagnosis and start of treatment (25.9 ± 30.2 days vs. 32.79 ± 32.51 days, *p* < 0.001), and greater nodal ratio of positive nodes to nodes examined (0.323 ± 0.364 vs. 0.176 ± 0.270, *p* < 0.0001; Table 1). A higher proportion of patients that were alive at 90 days also received neoadjuvant radiation therapy (16% vs. 10%, *p* < 0.001) and chemotherapy (21% vs. 14%, *p* < 0.001), had smaller tumor sizes (64.26 ± 125.86 mm vs. 79.41 ± 151.99 mm, *p* < 0.001), and lived in urban areas (56% vs. 51%, *p* < 0.001) with a median household income ≥ $63,000 for their residential area (32% vs. 27%, *p* < 0.001). Other differences between patient groups are highlighted in Table 1.

Ninety-day mortality varied based on type of surgery; 16,352 patients underwent total gastrectomy (1352, 8.3% 90-day mortality), 8160 patients underwent distal gastrectomy (659, 8.1% 90-day mortality), 3972 patients underwent en bloc gastrectomy (448, 11.3% 90-day mortality), and 10,669 patients underwent partial gastrectomy (1014, 9.5% 90-day mortality). Variations in 90-day mortality were also seen based on facility type, with 6.7% (1179/17,472) 90-day mortality in community cancer programs, 13.6% (319/2349) in comprehensive community cancer programs, 3.6% (1422/39108) in academic/research programs (including NCI-designated comprehensive cancer centers), 9.9% (532/5374) in Integrated Network Cancer Programs, 3.0% (21/705) in Unspecified facility types.

### AutoML can be feasibly used for predicting 90-day mortality

**Figure 1:**
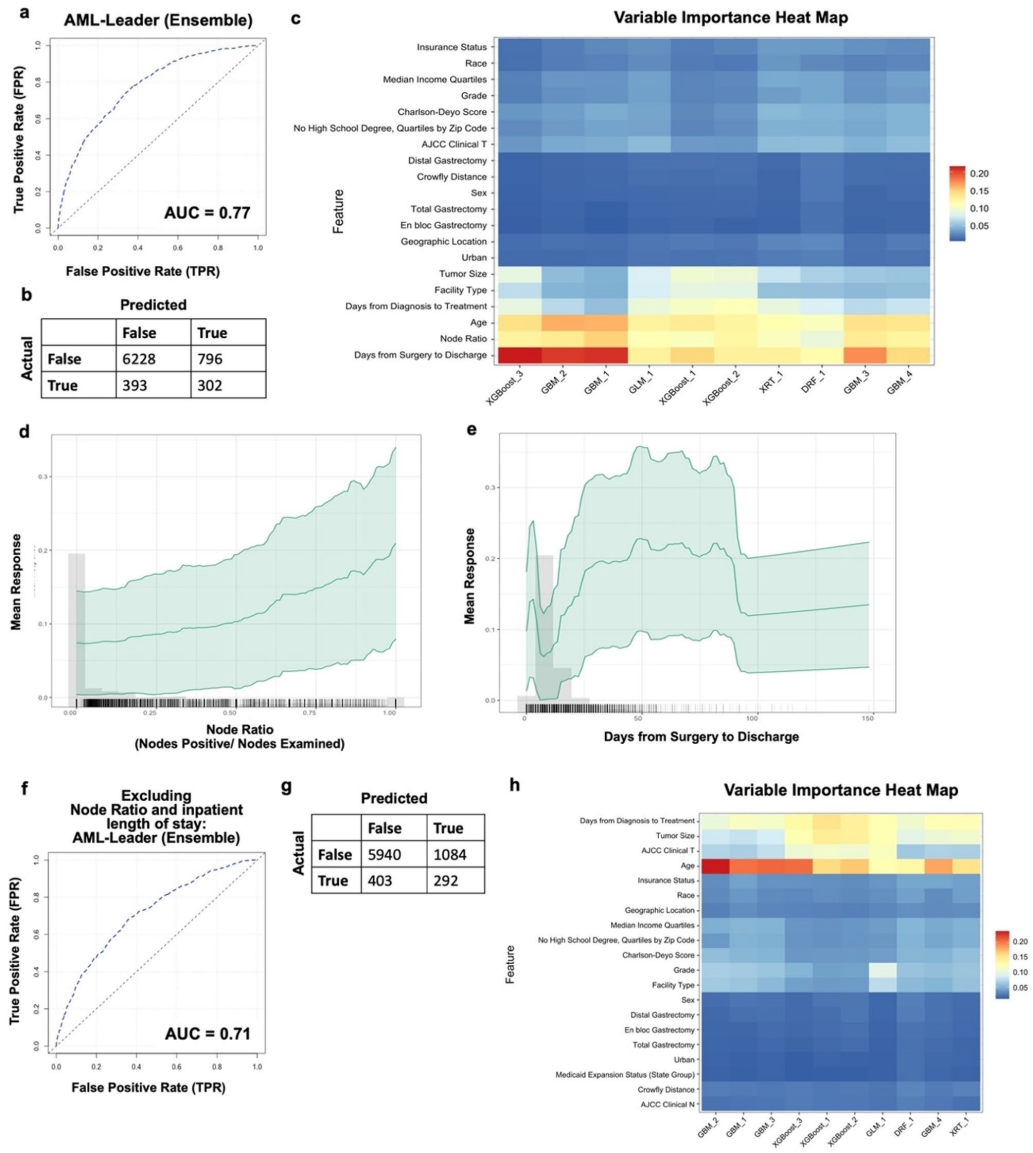
Exploratory prediction of 90-day mortality using AutoML. (**a**) Receiver operating curve and (**b**) F1 optimized confusion matrix showing the performance of leading ensemble model. (**c**) Variable importance heat map highlighting variables that were most influential for 90-day mortality prediction within each model generated. (**d**, **e**) partial dependency plots highlighting the importance of length of surgical inpatient stay and nodal ratio of positive nodes:nodes examined in predicting 90-day mortality. *The plateau seen within length of surgical inpatient stay represents patients that were alive and still admitted to the hospital after 90 days. (**f**) Receiver operating curve, (**g**) F1 optimized confusion matrix, and (**h**) variable importance plot for prediction of 90-day mortality without including length of surgical inpatient stay and nodal information in the model. Figures generated using H2O-R package version 3.40.0.4 (https://docs.h2o.ai/h2o/latest-stable/h2o-r/docs/articles/h2o-r-package.html).

To assess if 90-day mortality can be predicted using H2O.ai AutoML, 10 models were fit with 31,396 patients in the training set and 7712 in the validation set. The best performing model was a stacked ensemble (fivefold cross validation AUC 0.78; model performance on validation set AUC 0.77; Fig. 1A). The ensemble, when optimized based on F1 threshold (0.34), had a positive predictive value of 28%, negative predictive value of 94%, sensitivity of 43%, and specificity of 89% (Fig. 1B). Patient age, nodal ratio, and length of inpatient stay days since surgery were the three most influential variables across models (Fig. 1C). Partial dependency plots show that greater nodal ratio and longer inpatient stay (up to 90 days) greatly influenced model prediction (Fig. 1D,E).

Prediction of 90-day mortality preoperatively can not only inform patient prognosis, but also help improve patient selection for surgery. Thus, we created models without nodal ratio or inpatient length of stay. Performance of the leading ensemble declined (fivefold cross validation and model performance on validation set AUC 0.71; Fig. 1F), and when optimized based on F1 threshold (0.29), the model had a positive predictive value of 21%, negative predictive value of 94%, sensitivity of 42%, and specificity of 85% (Fig. 1G). Patient age remained highly influential in model prediction, along with clinical disease burden and time from diagnosis to treatment (Fig. 1H).

### Inclusion of predicted length of stay partially improves AutoML model prediction of 90-day mortality

**Figure 2:**
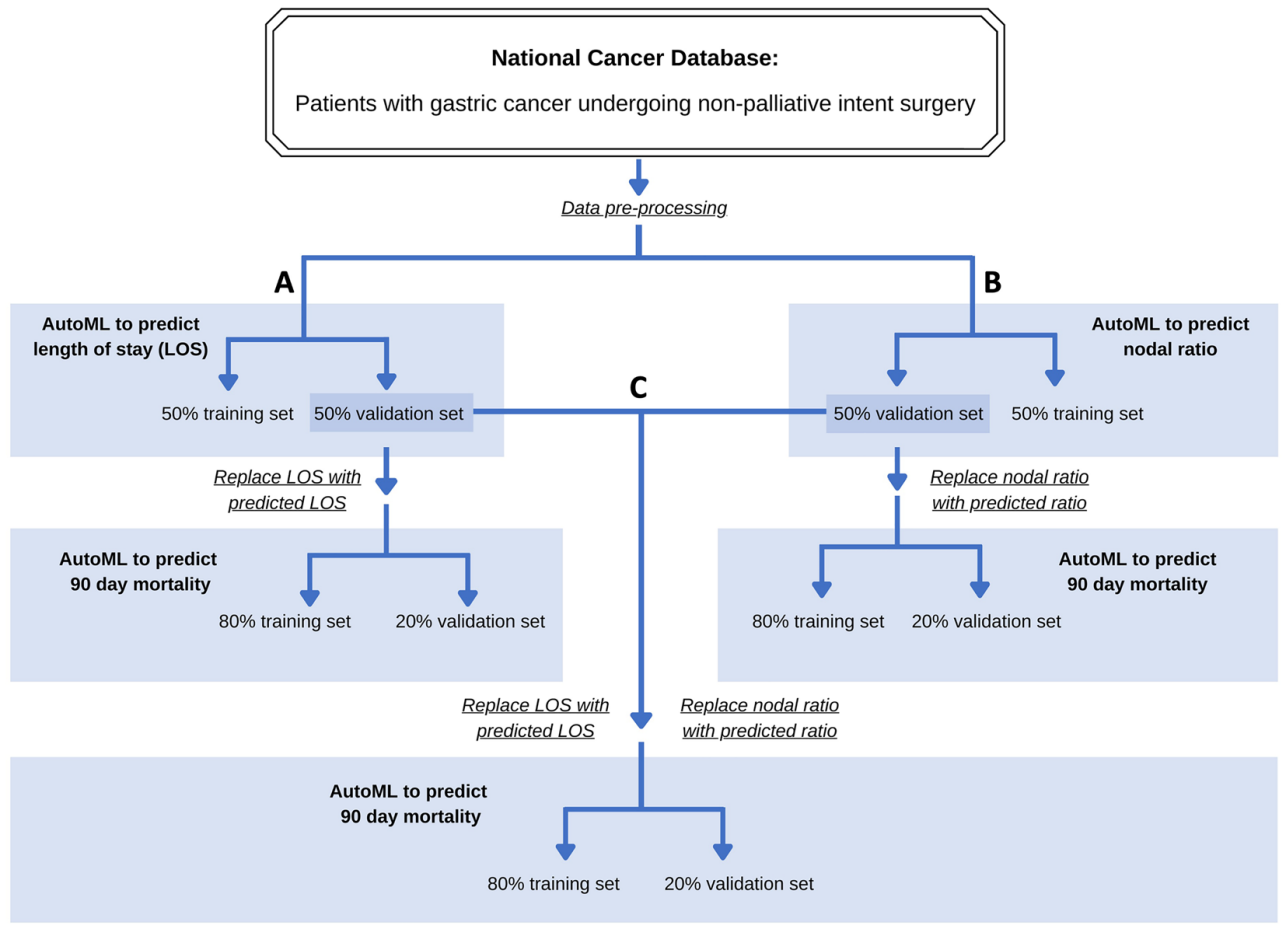
Multi-layered model-workflow. Multi-layered workflows that first predicts postoperative characteristics (A-length of stay; B-nodal ratio) and uses these predicted values to then predict 90-day mortality. Workflow C uses both predicted length of stay and nodal ratio. These multi-layered models allow for preoperative prediction of patients that are at risk for early postoperative mortality.

Given the importance length of stay had on model performance, we assessed whether a two-step approach could be used where: (1) prediction of patient length of stay using preoperative features (pLOS) followed by (2) prediction of 90-day mortality using input features that included pLOS (Fig. 2A). Performance of AutoML for predicting LOS is shown in Supplemental Fig. 1. Variables most important for predicting length of stay included patient’s income quartile, distal or en bloc gastrectomy, and race.

**Figure 3:**
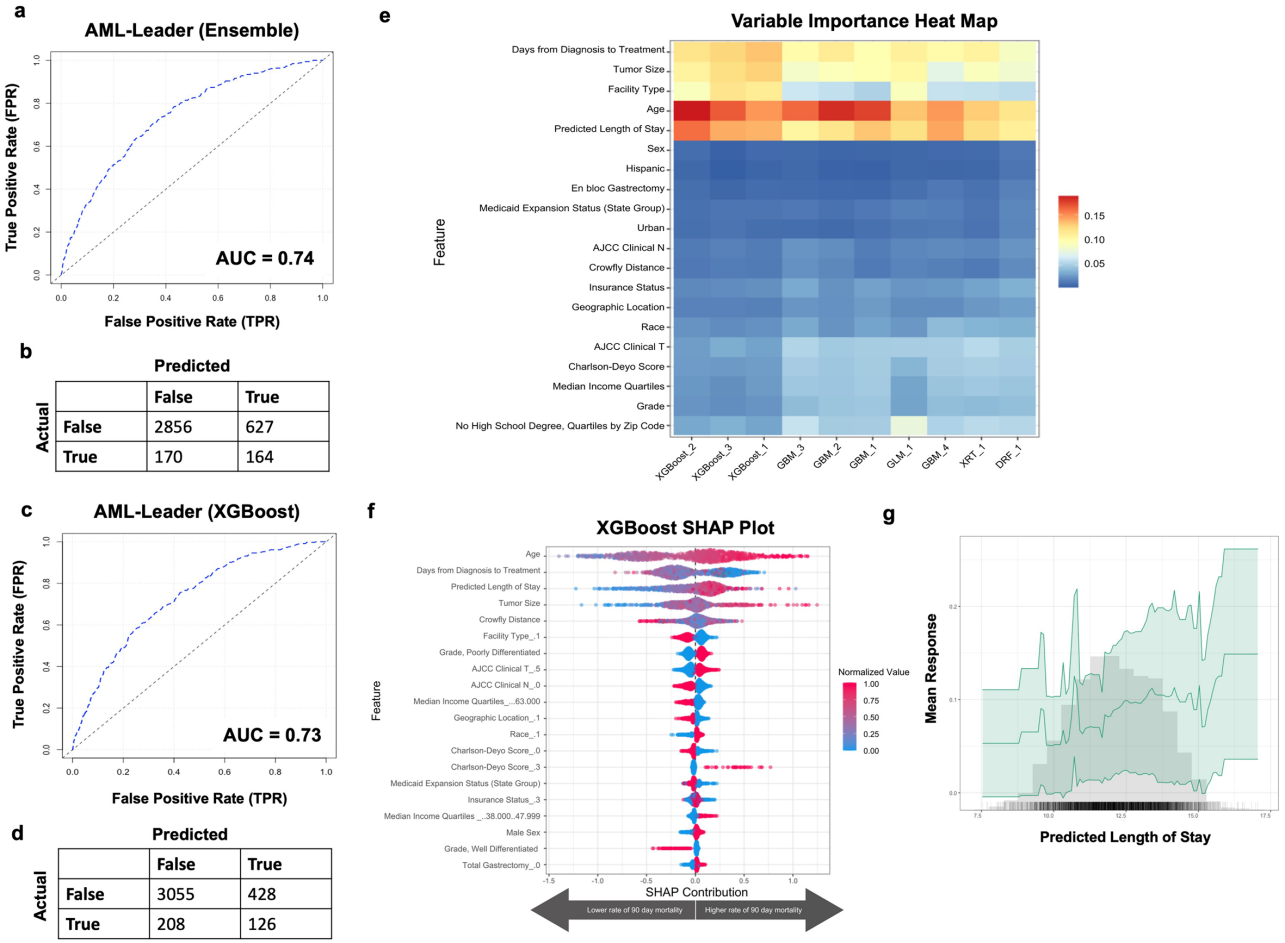
AutoML model prediction of 90-day mortality with predicted length of stay. (**a**) Receiver operating curve and (**b**) F1 optimized confusion matrix showing the performance of leading ensemble model. (**c**) Receiver operating curve and (**d**) F1 optimized confusion matrix of leading XgBoost model. (**e**) Variable importance heat map highlighting variables that were most influential for 90-day mortality prediction within each model generated. (**f**) Shapley additive explanations plot for leading Xgboost model. Variables of importance are ranked in descending order; within each variable, pink dots represent higher values, while blue dots represent lower values. (**g**) Partial dependency plot highlighting the importance of predicted length of surgical inpatient stay in predicting 90-day mortality. Figures generated using H2O-R package version 3.40.0.4 (https://docs.h2o.ai/h2o/latest-stable/h2o-r/docs/articles/h2o-r-package.html).

The pLOS values were then added as an additional input feature for predicting 90-day mortality. 10 models were tested, and the best performing models were a stacked ensemble (fivefold cross validation AUC 0.69 and model performance on validation set AUC 0.74; Fig. 3A) and XGboost (fivefold cross validation AUC 0.69 and model performance on validation set AUC 0.73; Fig. 3C). The ensemble, when optimized based on F1 threshold (0.29), had a positive predictive value of 21%, negative predictive value of 94%, sensitivity of 49%, and specificity of 82% (Fig. 3B). The XGboost model, when optimized based on a F1 threshold of 0.28, had a positive predictive value of 23%, negative predictive value of 94%, sensitivity of 38%, and specificity of 88% (Fig. 3D). The variables that were most influential for predicting 90-day mortality in this multi-layered model included older age, longer pLOS, lower time from diagnosis to treatment, and larger tumor size (Fig. 3E,F). Partial dependency plot for the pLOS confirmed that longer pLOS greatly influenced prediction of 90-day mortality (Fig. 3G).

### Inclusion of predicted nodal ratio partially improves AutoML model prediction of 90-day mortality

**Figure 4:**
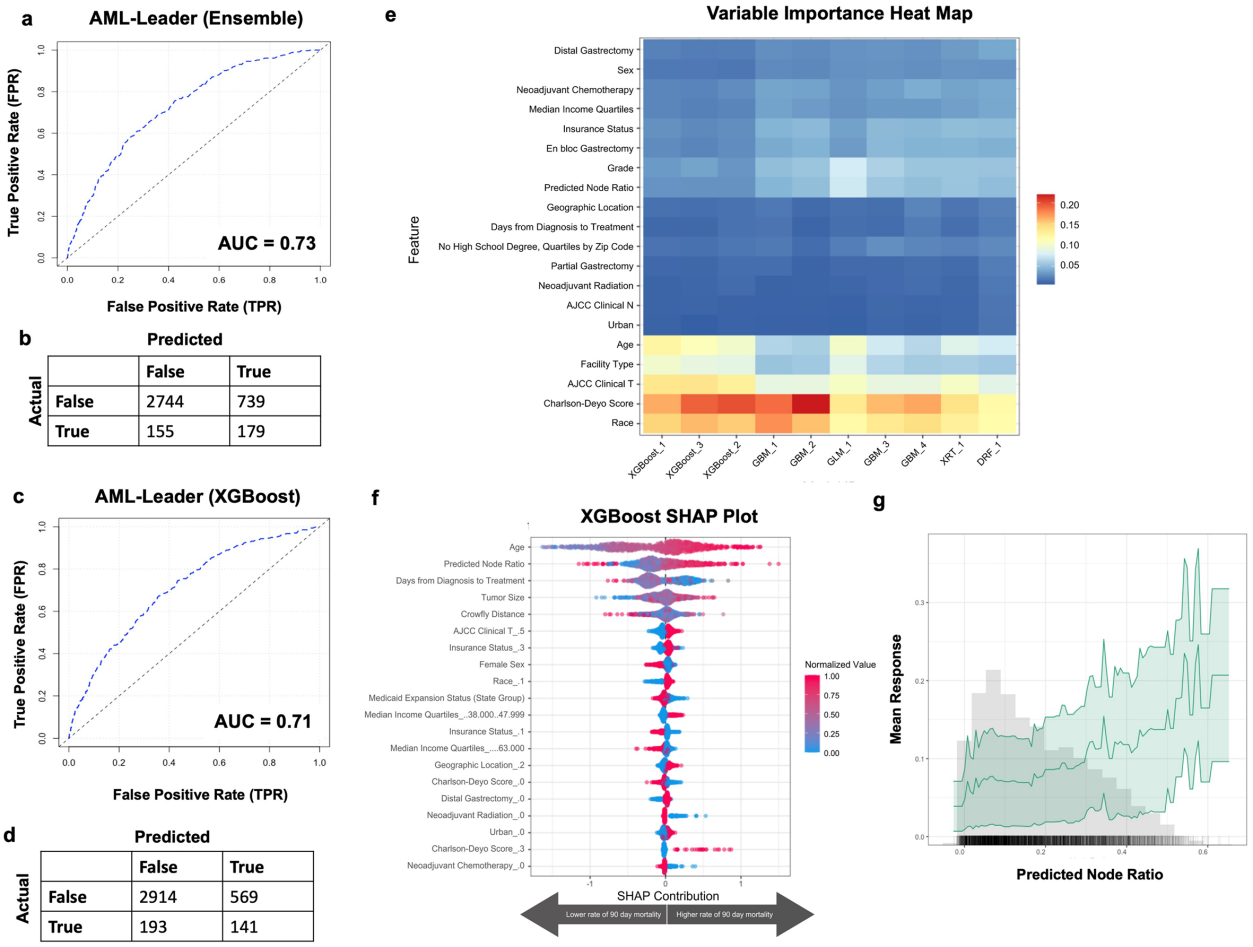
AutoML model prediction of 90-day mortality with predicted nodal ratio. (**a**) Receiver operating curve and (**b**) F1 optimized confusion matrix showing the performance of leading ensemble model. (**c**) Receiver operating curve and (**d**) F1 optimized confusion matrix of leading XgBoost model. (**e**) Variable importance heat map highlighting variables that were most influential for 90-day mortality prediction within each model generated. (**f**) Shapley additive explanations plot for leading Xgboost model. Variables of importance are ranked in descending order; within each variable, pink dots represent higher values, while blue dots represent lower values. (**g**) Partial dependency plot highlighting the importance of predicted nodal ratio in predicting 90-day mortality. Figures generated using H2O-R package version 3.40.0.4 (https://docs.h2o.ai/h2o/latest-stable/h2o-r/docs/articles/h2o-r-package.html).

Given that the inclusion of pLOS only partially improved model prediction of 90-day mortality, we tested whether inclusion of predicted nodal ratio improved performance (Fig. 2B). Performance of AutoML for predicting nodal ratio is shown in Supplemental Fig. 2. The pNodeRatio values were then used as an additional input feature for predicting 90-day mortality. 10 models were tested, and the best performing model was a stacked ensemble (fivefold cross validation AUC 0.70 and model performance on validation set AUC 0.73; Fig. 4A). The best performing XGboost model had an AUC of 0.68 on fivefold cross validation AUC of 0.71 when tested on validation set (Fig. 4C). The ensemble, when optimized based on F1 threshold (0.29), had a positive predictive value of 19%, negative predictive value of 95%, sensitivity of 54%, and specificity of 79% (Fig. 4B). The XGboost model, when optimized based on F1 threshold (0.27), had a positive predictive value of 20%, negative predictive value of 94%, sensitivity of 42%, and specificity of 84% (Fig. 4D). The variables that were most influential for predicting 90-day mortality in this multi-layered model included older age, pNodeRatio, and clinical disease burden (Fig. 4E,F). Partial dependency plot for the pNodeRatio confirmed that higher nodal ratio greatly influenced prediction of 90-day mortality (Fig. 4G).

### Inclusion of both predicted length of stay and nodal ratio does not further improve AutoML model prediction of 90-day mortality compared to models with either variable along

**Figure 5:**
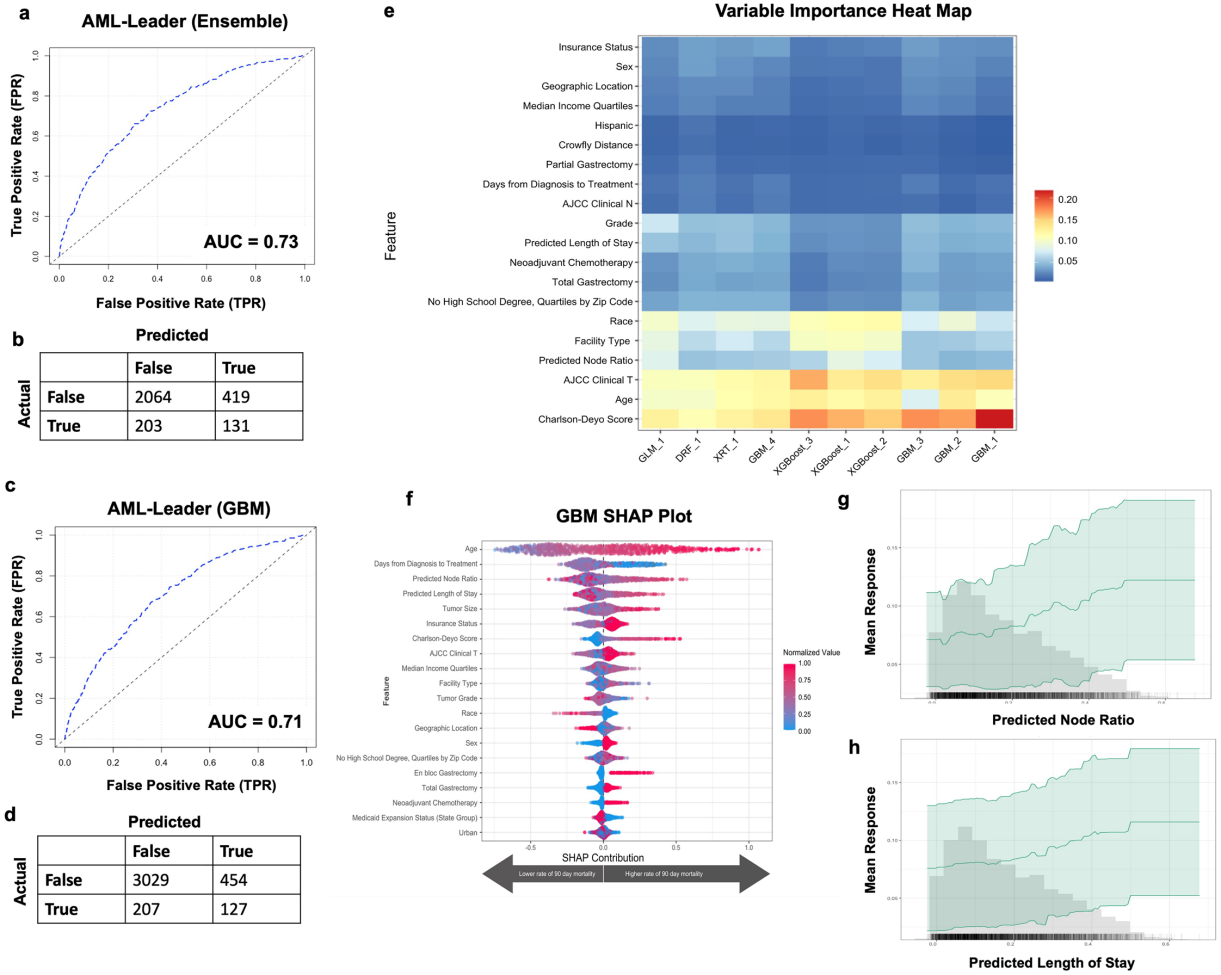
AutoML model prediction of 90-day mortality with both predicted length of stay and predicted nodal ratio. (**a**) Receiver operating curve and (**b**) F1 optimized confusion matrix showing the performance of leading ensemble model. (**c**) Receiver operating curve and (**d**) F1 optimized confusion matrix of leading gradient boosting model (GBM). (**e**) Variable importance heat map highlighting variables that were most influential for 90-day mortality prediction within each model generated. (**f**) Shapley additive explanations plot for leading gradient boosting model. Variables of importance are ranked in descending order; within each variable, pink dots represent higher values, while blue dots represent lower values. (**g**, **h**) Partial dependency plots highlighting the importance of predicted length of stay and predicted nodal ratio in predicting 90-day mortality. Figures generated using H2O-R package version 3.40.0.4 (https://docs.h2o.ai/h2o/latest-stable/h2o-r/docs/articles/h2o-r-package.html).

Given incremental improvements in 90-day mortality prediction with models that either had pLOS or pNodeRatio, we next included both predicted variables as input features Fig. 2C). This approach did not significantly improve model performance (leading ensemble AUC of 0.73 on validation set, and leading gradient-boosting model AUC of 0.71; Fig. 5A,C). The ensemble, when optimized based on F1 threshold (0.29), had a positive predictive value of 24%, negative predictive value of 91%, sensitivity of 39%, and specificity of 83% (Fig. 5B). The gradient boosting model, when optimized based on F1 threshold (0.28), had a positive predictive value of 22%, negative predictive value of 94%, sensitivity of 38%, and specificity of 87% (Fig. 5D). Both increased pNodeRatio and higher pLOS were influential in predicting 90-day mortality (Fig. 5E-H).

Finally, we completed sensitivity analyses stratified by facility type and surgical approach, given the aforementioned heterogeneity in 90-day mortality based on these factors (Supplemental Fig. 3). Model performance was maintained across facility type and surgical approach.

## Discussion

The major findings of this study are: (1) in a cohort of stage I–III gastric cancer patients that underwent gastrectomy, AutoML performed well in predicting early postoperative mortality; (2) the generated AutoML models produced predictions that could help with clinical patient prognostication and counseling of those predicted to be high risk; (3) the variables most influential in predicting 90-day mortality include older age, high nodal ratio of positive nodes to nodes examined, and prolonged hospital length of stay following surgery; (4) a multi-step approach that first predicts a postoperative characteristic (i.e. pLOS and pNodeRatio) and then 90-day mortality can be used to design models for preoperative use. Our work shows that AutoML can be feasibly, efficiently, and easily be used for training and validating ML models using commonly collected perioperative factors. To our knowledge, our study is the first to demonstrate the applicability of AutoML for early postoperative mortality prediction in cancer surgery. Thus, in addition to its potential utility for surgical treatment of patients with gastric cancer, our study supports broader evaluation and application of AutoML to guide surgical oncologic care.

Numerous studies have highlighted the importance of predicting mortality among patients with advanced cancers to assist with appropriate treatment planning and patient counseling^3,4,18^. Post-gastrectomy outcomes and mortality have been associated with several factors including stage of the disease, lymph node metastases, co-morbidities from neoadjuvant therapy, and age of the patient^2,19–21^, but few clinical support tools or algorithms have been developed to accurately inform patient prognostication based on perioperative variables. Niu et al.’s review on the application of artificial intelligence within gastric cancer highlights several studies that used ML models to diagnose gastric cancer and predict recurrence and metastasis; however, most of these studies utilized endoscopy or computed tomography images, pathology slices, or genetic features^7^. Image-based prediction models require large quantities of accurately annotated data^7,22^, and acquiring genetic features for all patients adds to the cost of patient care and requires substantial time. One of the most widely used surgical risk calculators was developed by the American College of Surgeons National Surgical Quality Improvement Program (ACS NSQIP). The ACS NSQIP risk calculator previously has been studied for the purpose of predicting mortality following gastrectomy. In comparison to our reported models, the ACS NSQIP risk calculator shows similar, and sometimes worse, performance in predicting mortality in this population^23^. An advantage to our approach is the inclusion of cancer-specific variables including staging, receipt of preoperative oncologic therapies, and tumor characteristics. Furthermore, Lu et al.’s systemic review of 15 articles that utilized ML models to predict early mortality in patients with cancer using electronic health record data showed that model performance ranged from AUCs of 0.71 to 0.92^24^. Unlike those studies, we utilized common data elements found within readily available real world data sources to train our ML models in patients with gastric cancer that underwent non-palliative gastrectomy. While many prior studies of ML models rely on small sample sizes, our study with 39,108 patients highlights promising abilities of AutoML models to predict early-mortality among cancer patients using data from population-level registries. Our approach provides a template for developing cost-effective and easy-to-implement decision-support tools for guiding patient selection for surgical treatment in this population.

Our use of an interpretable machine learning approach facilitates the identification of potentially targetable risk factors. Older patient age, higher nodal ratio, and greater number of days between surgery and discharge were the three most influential variables across models in predicting 90-day mortality. This is consistent with Shannon et al.’s multivariate retrospective analysis of patients within NCD with stage I–III gastric adenocarcinoma that underwent total gastrectomy; their results showed that increasing age and a lower number of lymph nodes examined are associated with 90-day mortality^2^. Shu et al. further showed that older age (> 70 years) was associated with increased rate of complications (20% vs. 11% in those < 70 years), and higher 90-day mortality (3.7% vs. 0.5%) in a cohort of 534 patients at a single-institution. Notably, age independently predicted mortality after controlling for tumor biology, cancer stage, adjuvant therapy, and postoperative complications^25^, thereby highlighting the need for careful evaluation and counseling of older patients prior to gastrectomy.

For ensuring clinical utility, the timing of implementing predictive models is crucial. The initial model in this study can inform postoperative patient prognostication and highlighted the importance of postoperative length of stay and nodal ratio in predicting 90-day mortality. This is consistent with previous efforts to enhance prognostication in gastric cancer which reported that the number of nodes examined and nodal positivity independently influence survival in gastric cancer^26,27^. However, preoperative prediction is necessary to assist with both patient prognostication and selection of surgery. To ensure that our predictive models are useful in the preoperative setting, we used a multi-step modeling strategy where we first predicted length of stay and nodal ratio only using parameters available preoperatively. These predicted features were then used as input features in our final model for predicting mortality, which showed high discriminatory capability. This complex strategy was easy to implement through H2O.ai’s AutoML tools.

Despite better performance in prediction of pNodeRatio compared to pLOS, inclusion of pLOS provided the most improvement in model performance in predicting 90-day mortality. This suggests that patients that are at higher risk for longer hospital stays are highly susceptible to early postoperative mortality. Our work highlighted that patient’s income quartile, undergoing distal or en bloc gastrectomy as well as racial background influenced length of stay predictions. This is in-line with prior studies that show that the extent of resection and type of surgical procedure are independently predictive of postoperative length of stay in patients with gastric cancer^28^. In addition to these factors, patients’ preoperative physical function/strength and co-morbidities influence both postoperative complications and length of hospital stay^29,30^. Future models that incorporate these preoperative characteristics may enhance pLOS prediction and subsequent early mortality prediction. Importantly, the congruence between prior research and the variables that were most influential in AutoML models provide confidence in these models’ clinical utility.

The influence of hospital length of stay on predicting early mortality also provides an opportunity for implementing clinical programs that help reduce this duration, to then potentially reduce early postoperative mortality. Enhanced Recovery After Surgery (ERAS) protocols have been implemented following gastrectomy^31,32^, and they incorporate preoperative counseling and nutrition, earlier mobilization and feeding following surgery, avoidance of abdominal drains, and nasogastric/nasojejunal decompression^33,34^. Wee et al.’s meta-analysis comparing conventional postoperative care versus ERAS protocols showed that ERAS programs decreased length of stay and care costs but did not significantly alter 30-day postoperative mortality or postoperative morbidity^33^. Weindelmayer’s single-institution study of 351 gastric cancer patients reported a reduction in 90-day mortality among patients in the ERAS program (0.8% vs. 4.8% control); however, their overall 90-day mortality was only 2%^35^. Further research is necessary to optimize ERAS programs and to assess whether they reduce early postoperative mortality. Within our dataset, there was a cohort of patients that were still admitted to the hospital past 90 days postoperatively, and while the primary aim of this study was to assess early mortality, further research is necessary to understand predictors of prolonged hospital stays as well as morbidity, mortality, and quality of life outcomes among these populations.

Numerous studies have piloted clinical implementation of machine learning tools. Avati et al. developed a deep neural network that screens electronic health records from of all admitted patients at Stanford Hospital and predicts all-cause mortality within 3–12 months. They implemented the ML algorithm as a screening tool that notifies palliative care of positive predictions^36^, thereby streamlining patient-referrals and demonstrating how ML-based early mortality predictions can improve the efficiency of patient care. Manz et al. developed an ML-algorithm to predict 180-day mortality among oncology clinic patients within a health system in Pennsylvania. Their randomized clinical trial implementing this model along with behavioral nudges (weekly performance feedback to clinicians) showed increased rates of serious illness conservations with high mortality risk patients—a positive clinician behavior that improves end-of-life care^22^. Our results provide the necessary first step towards bedside application by demonstrating the feasibility of using AutoML to produce robust mortality predictions. Specifically, AutoML-based predictions could be used to augment perioperative risk stratification and postoperative treatment planning. Models can be implemented through direct integration with electronic health records as well as through development of websites/applications (as done with NSQIP risk calculators) for bedside use. Future work will focus on developing these strategies for implementation of the model developed in this study.

A crucial point to emphasize is that we do not advocate for strictly following the output of our model-derived prediction to make clinical decisions. Frequently, clinicians, patients, and caregivers are faced with difficult conversations to decide on the optimal treatment trajectory to pursue. Estimating surgical risk using clinical factors and surgeon judgment is frequently part of these discussions—even in the absence of ML tools. How AutoML can be useful in these circumstances is through augmentation and providing another parameter to help inform shared decisions.

Our results must be interpreted while considering the limitations. While NCDB allows us to train ML models on a large cohort of heterogenous patients, the database itself is limited by missing data^37^, lack of information on the cause of death, and biases introduced by retrospective analysis^2^. Additionally, the database does not include information on patient transfers to hospice care, so we cannot discern what proportion of patients underwent hospice deaths. While our results were consistent with prior work that utilized NCDB^2^, the nearly 9% 90-day mortality seen in our patient cohort is higher compared to other series^38,39^. Although, the other studies had smaller sample sizes and less heterogeneity in treatment centers. Given our goal of making this model broadly applicable, we elected to include all types of gastric resection and type of center where surgery was performed. As expected, 90-day mortality greatly varied across resection and center types. Our sensitivity analysis showed similar model performance in low mortality centers (academic)/ resection types (total/distal gastrectomy). Nonetheless, prior to clinical implementation, models should be validated and optimized based on institutional data; this process is simplified given the easy-to-use nature of the AutoML platform. Finally, while NCDB captures approximately 70% of cancer patients, it only has data from patients that were treated at accredited CoC facilities, and thus is not generalizable to the entire US population^2,37^. Nonetheless, AutoML is able to handle missing data and reasonably predict early mortality in this heterogenous population using only the available features. Our work only focused on one AutoML approach, and further studies are necessary to understand the applicability of other models within surgical risk prediction. Lastly, while we focused on mortality prediction, it is not the only outcome of interest for patients and families considering gastric surgery. Thus, future studies focused on morbidity and quality of life predictions are needed.

## Conclusion

While surgery is the only curative therapy for patients with gastric cancer, 90-day postoperative mortality remains high, and prediction of early postoperative mortality is challenging. While machine learning algorithms have the potential to make predictions more accurately when compared to traditional statistical methodologies, the need for computational and statistical expertise has been suggested to limit the widespread application of machine learning within healthcare. In a large cohort of gastric cancer patients that underwent gastrectomy, our study shows that AutoML performs well in predicting mortality. Models can further be optimized for preoperative prediction, thereby not only allowing for robust patient prognostication but also informing patient selection for surgery. We were also able to identify key perioperative variables that were influential in outcome prediction to guide future interventions that mitigate risks of early postoperative mortality. Our work provides a framework for effective, scalable, easy-to-implement, and explainable machine learning to inform clinical decision-making.

## Supplementary Information

Supplementary Figures.

## Data Availability

The datasets generated and/or analyzed during the current study are available in the National Cancer Database Participant Use Files for eligible users: https://www.facs.org/quality-programs/cancer-programs/national-cancer-database/puf.
