# Supplementary material for: Automated machine learning (AutoML) can predict 90-day mortality after gastrectomy for cancer
Source: Sci Rep. 2023 Jul 8;13:11051. doi: 10.1038/s41598-023-37396-3 (PMC10329647; doi:10.1038/s41598-023-37396-3)
Supplement: Supplementary file 1 — Supplementary Figures. [file 41598_2023_37396_MOESM1_ESM.docx]

**SUPPLEMENTAL FIGURES**

**
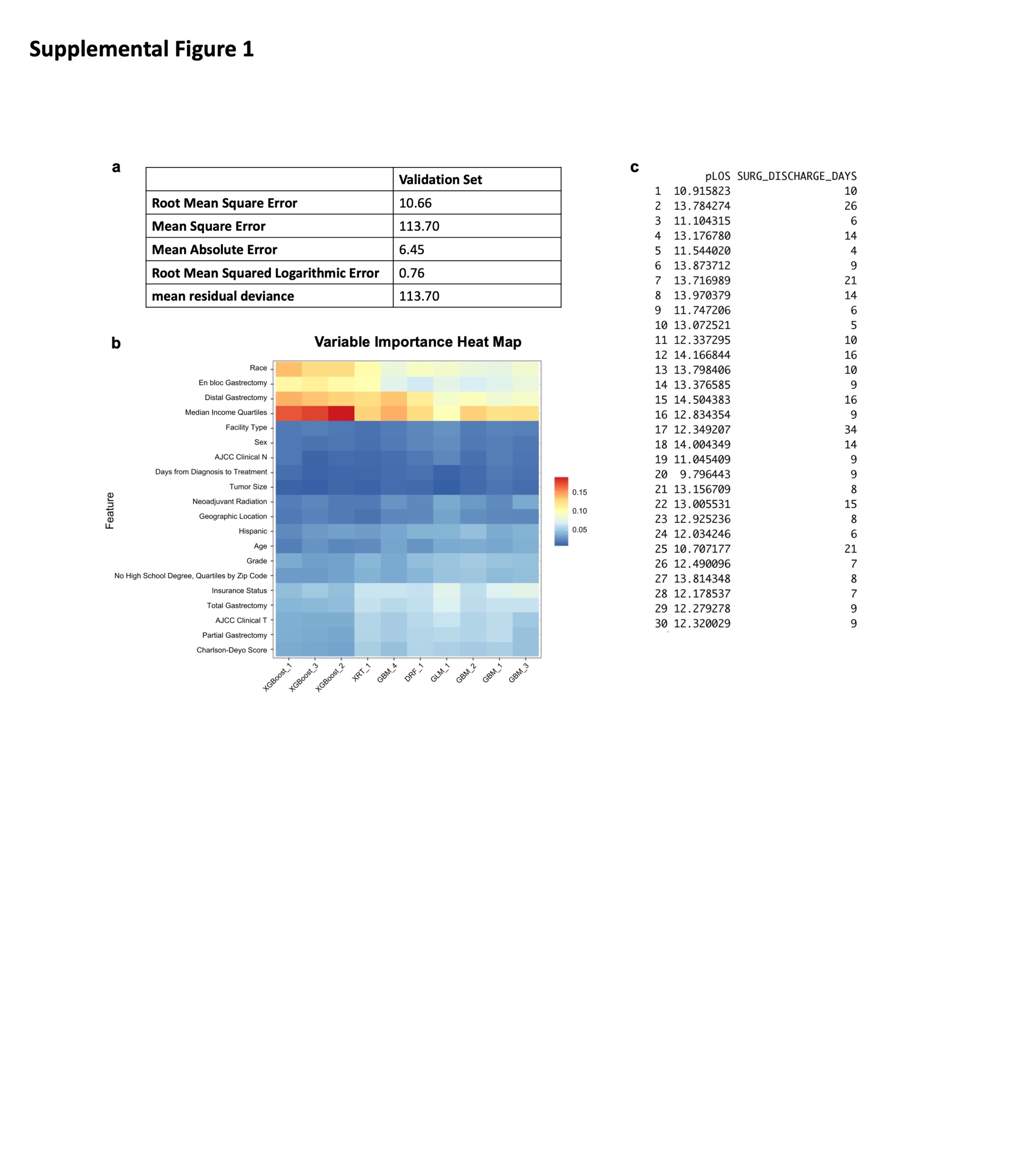
**

**Supplemental Figure 1. AutoML model prediction of inpatient post-operative length of stay.** (a) Model performance, (b) variable importance heat map highlighting variables that were most influential for length of stay prediction, and (c) representative predicted length of stay (pLOS) and original values (SURG_DISCHARGE_DAYS).


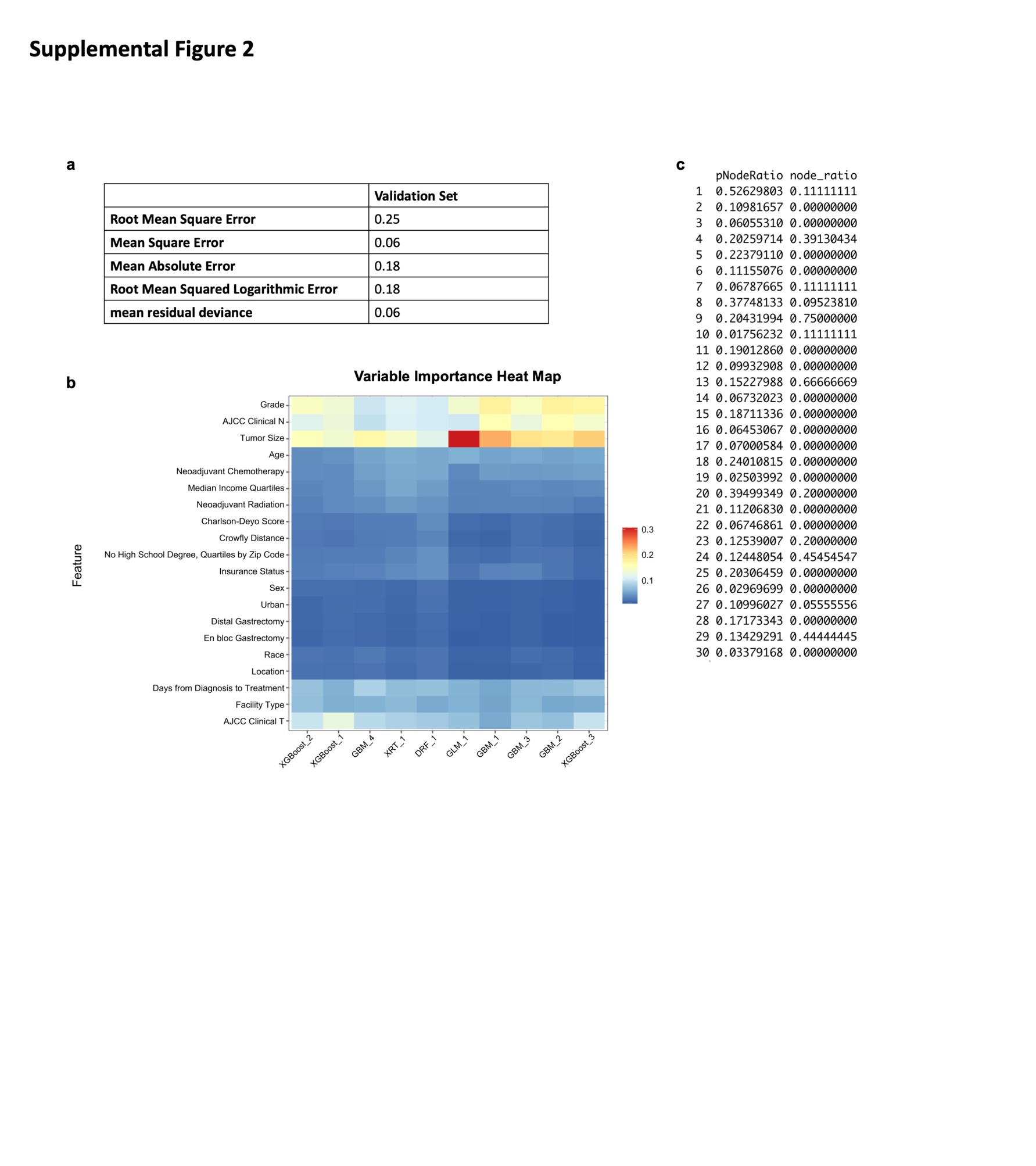


**Supplemental Figure 2. AutoML model prediction of nodal ratio.** (a) Model performance, (b) variable importance heat map highlighting variables that were most influential for nodal raio prediction, and (c) representative predicted node ratio (pNodeRatio) and original values (node_ratio).


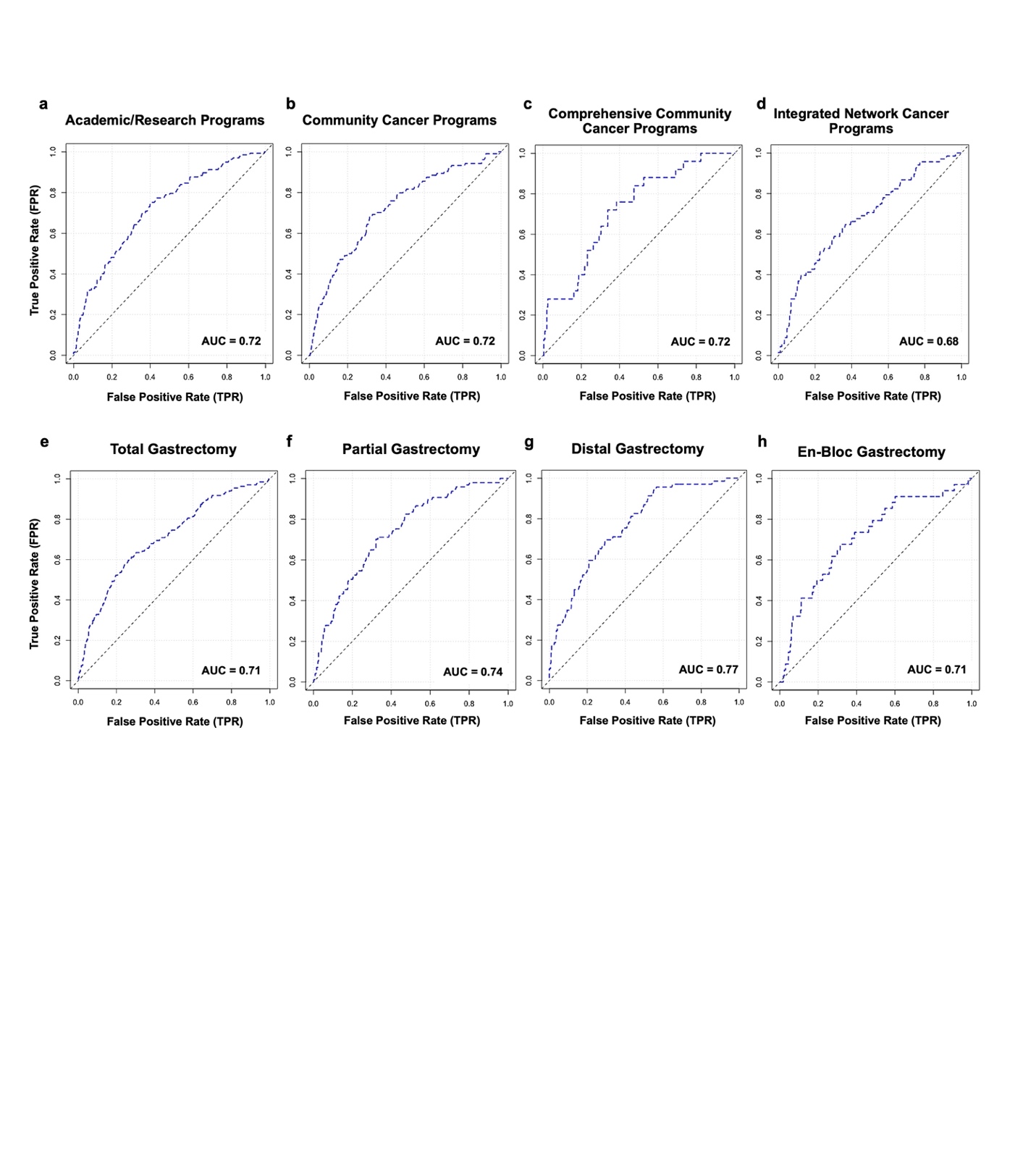


**Supplemental Figure 3. AutoML model prediction of 90-day mortality across center types where surgery was performed and gastrectomy types.** Receiver operating curve showing performance of leading ensemble model within patients receiving care at (a) academic/research program, (b) community cancer program, (c) comprehensive community cancer program, and (d) integrated network cancer programs, and undergoing (e) total gastrectomy, (f) partial gastrectomy, (g) distal gastrectomy, and (h) en-bloc gastrectomy.
